# MMBs_TransNeXt: a hierarchical adaptive feature learning model for pig behavior recognition in precision livestock farming

**DOI:** 10.3389/fvets.2026.1904986

**Published:** 2026-09-03

**Authors:** Meng Han, Wangli Hao, Hao Shu, Xinyuan Hu, Qingqing Li, Fuzhong Li

**Affiliations:** 1 School of Information Science and Engineering, Shanxi Agricultural University, Jinzhong, China; 2 Faculty of Software Technologies, Shanxi Agricultural University, Jinzhong, China

**Keywords:** Bidirectional Modulation Feature Fusion, cross-level feature interaction, Multi-scale Detail Fusion Convolution, Multi-stage Adaptive Feature Fusion, pig behavior recognition, precision livestock farming

## Abstract

Accurate recognition of pig behavior plays a critical role in enhancing animal welfare and management efficiency for precision livestock farming. Nevertheless, existing methods are commonly limited by rigid feature fusion strategies, insufficient preservation of spatial details, and weak interactions across hierarchical features, which severely compromise recognition performance in complex farm scenarios. To address these challenges, we present a novel deep learning architecture termed MMBs_TransNeXt for pig behavior recognition. This model establishes a unified, hierarchical and adaptive feature learning framework. It employs Multi-stage Adaptive Feature Fusion (MAFF) to adaptively integrate semantic and fine-grained information across different network stages. It enhances the representation of subtle behavioral traits through Multi-scale Detail Fusion Convolution (MDFC). Meanwhile, it facilitates effective information communication and interaction among multi-level features via Bidirectional Modulation Feature Fusion (BMFF). Extensive experiments on a real-world pig behavior dataset validate that MMBs_TransNeXt attains state-of-the-art performance with 95.77% accuracy, substantially outperforming representative CNN and Transformer baselines. This work offers a robust technical foundation for intelligent visual monitoring and welfare evaluation in modern precision pig farming systems.

## 1 Introduction

Pig behavior recognition is a critical component of modern precision livestock farming systems (1). Accurate behavioral analysis not only quantifies animal welfare indicators but also enables data-driven optimization of environmental parameters and nutritional strategies (2, 3), thus enhancing production efficiency and promoting the sustainable development of animal agriculture (4). Despite its significance, automated pig behavior recognition remains challenging. In complex farm scenarios, behavioral differences are often subtle and are easily affected by factors such as overlapping and occlusion. These complex scene-related factors further increase the difficulty of achieving robust recognition (5).

Conventional pig behavior analysis relies on manual observation and sensor-based technologies (6). In contrast, manual annotation offers intuitive insights; however, its subjectivity and labor-intensive nature limit scalability (7, 8). On the other hand, while sensor technologies can accurately capture pig behavior characteristics (9), challenges with complex deployment and potential detachment issues can compromise data accuracy and pose risks to animals' health.

Multimodal sensing and adaptive feature processing are effective for complex monitoring across various livestock species. In pig behavior recognition, 3D data and multi-scale fusion are particularly important. Similar techniques have also been validated in cattle monitoring. Imamura et al. developed a 3D camera-based method for automated body condition scoring by extracting geometric features such as Root-Mean-Square Deviation and convex hull volume from depth images (10). Moe et al. proposed a comprehensive AI-powered visual system that integrated 22 multi-modal cameras and deep learning for non-invasive cattle health monitoring, achieving high accuracy in individual identification and lameness detection (11). Pretto et al. designed a low-cost visual ear tag recognition system based on YOLOv3 and optical character recognition technology, incorporating an error correction mechanism, which demonstrated satisfactory performance in individual identification and drinking behavior monitoring (12). Zhang et al. deployed an SSD MobileNet V2 model through TensorFlow.js for edge computing-based recognition of cattle rumination and eating behaviors, achieving desirable identification outcomes (13). Wu et al. developed a ResNet101-ASPP network for multi-scale dairy cow keypoint detection and simultaneously extracted seven motion features from tracked keypoint trajectories. Their method improved robustness under occlusion and scale variation and provided richer support for behavior analysis (14).

These studies emphasize adaptive feature extraction and non-invasive sensing, offering valuable references for optimizing pig-focused models. In particular, their insights into multi-scale representation, motion feature extraction, and non-invasive monitoring are especially relevant for addressing rigid feature fusion and detail loss in pig behavior recognition models.

Recent advancements in deep learning technology have enabled the automation of pig behavior recognition using convolutional neural networks (CNNs) (15, 16). Earlier CNN-based frameworks achieved this primarily by focusing on spatial feature extraction.

An et al. introduced ConvNeXt (17), which combined large convolutional kernels and inverted bottleneck structures, resulting in an accuracy of 87.8% on ImageNet and refining CNN architectures. Paudel et al. tackled the challenges of identifying individual pigs in large-scale farms by employing 3D spatial data-based deep learning solutions, achieving an F1-score of 91% under specific conditions (18). More recently, Elnady et al. combined YOLO and LSTM modules (19), demonstrating remarkable performance on public action recognition benchmarks. Additionally, Shuqin et al. developed a behavior recognition framework that integrates YOLOX-S and YOLOv5 detectors for monitoring large-scale pig farms, showing robust performance in field trials (20). Notably, Ma et al. proposed an improved YOLOv4 lightweight neural network (M-YOLOv4-C) with MobileNet-v3 as the backbone, achieving 98.15% mAP and 106.3 FPS for pig face recognition, providing technical support for individual pig management (21).

The emergence of Transformer architectures (22) has revitalized research on pig behavior recognition by offering enhanced capabilities to capture long-range dependencies in behavioral sequences. Dosovitskiy et al.'s Vision Transformer (ViT) (23) pioneered pure Transformer-based visual architectures, overcoming the limitations of convolutional locality using self-attention mechanisms, which facilitate long-term behavioral analysis in pigs. Liu et al. introduced the Swin Transformer (24), which employed shifted window self-attention and progressive feature map downsampling to achieve linear computational complexity, improving accuracy by 6.3% compared to ViT.

Additionally, Yang et al. created a multimodal fusion framework (TMF) (25) that innovatively integrated unimodal self-attention enhancement modules to capture global contextual features, achieving a superior accuracy in general behavior recognition. Moreover, Pu et al. combined MobileNetV2 with an enhanced Autoformer (26), demonstrating robust performance in practical farm scenarios. Furthermore, Shi introduced the TransNeXt model (27), which integrated aggregated attention and Convolutional Gated Linear Units (28), achieving 84.0% accuracy on ImageNet-1K while reducing parameters by 69%.

However, these current deep learning approaches still face limitations that hinder optimal performance in complex farm settings. Specifically, their feature fusion strategies are often static, leading to inflexible feature representation. The loss of spatial details in deep layers and weak interactions between features extracted at different hierarchical levels restrict their ability to robustly discern subtle behavioral differences. To overcome these limitations, this paper proposes a novel pig behavior recognition model named MMBs_TransNeXt.

The main contributions of this paper are summarized as follows:

- This paper introduces MMBs_ TransNeXt, a hierarchical adaptive feature learning architecture for accurate pig behavior recognition in precision livestock farming. The network unifies adaptive multi-stage feature fusion, multi-scale detail enhancement, and bidirectional cross-level feature interaction, which effectively alleviates the problems of rigid feature fusion, insufficient spatial detail preservation, and weak hierarchical feature communication existing in mainstream approaches.
- The Multi-stage Adaptive Feature Fusion (MAFF) mechanism is devised to dynamically integrate semantic information and fine-grained features across different network stages. By learning stage-adaptive weights, MAFF strengthens the complementation between shallow details and deep semantics, and enhances the discriminative representation of pig behavioral characteristics.
- A Multi-scale Detail Fusion Convolution (MDFC) module is proposed to suppress detail blurring caused by cross-channel interference. Equipped with a heterogeneous dual-branch structure, MDFC separately enhances local fine-grained feature extraction and global contour perception, thereby preserving subtle behavioral traits more effectively.
- The Bidirectional Modulation Feature Fusion (BMFF) mechanism is constructed to facilitate efficient information communication among multi-level features. By combining cross-modulation and self-modulation strategies, BMFF strengthens feature complementation and information flow across levels, further improving recognition accuracy and robustness in complex scenes.

## 2 Materials and methods

### 2.1 Dataset

**Figure 1:**
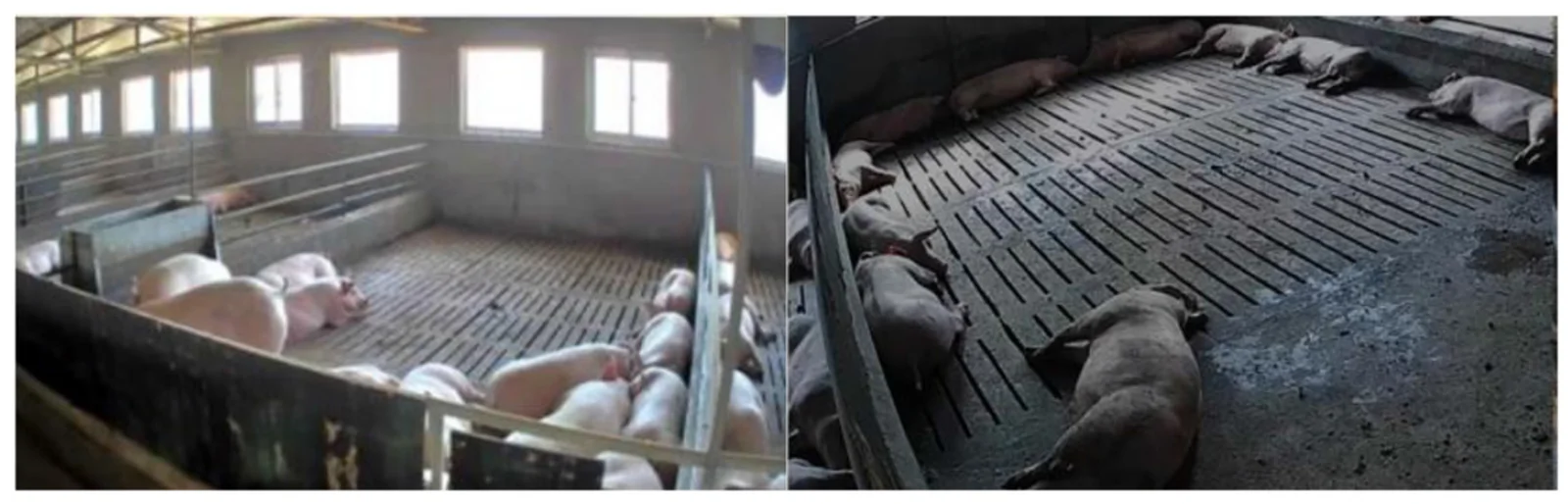
Representative camera views inside pig houses.

The pig behavior recognition data were collected at the pig breeding base of Nonglvyuan Agricultural Co., Ltd. in Xiangfen County, Linfen City, Shanxi Province, China. Data acquisition was conducted from August 12, 2022, to September 25, 2022. Six pig houses were utilized; each house housed ten six-month-old pigs. Hikvision DS-2DE3Q120MY-T/GLSE cameras were installed in all six houses. Each camera was mounted on the sidewall at a height of 3m and tilted at a 45° angle toward the pen. Video was recorded at a resolution of 1920 × 1080~pixels and a frame rate of 25 fps in the RGB color space. A representative camera view inside one of the pig houses is shown in Figure 1.

**Figure 2:**
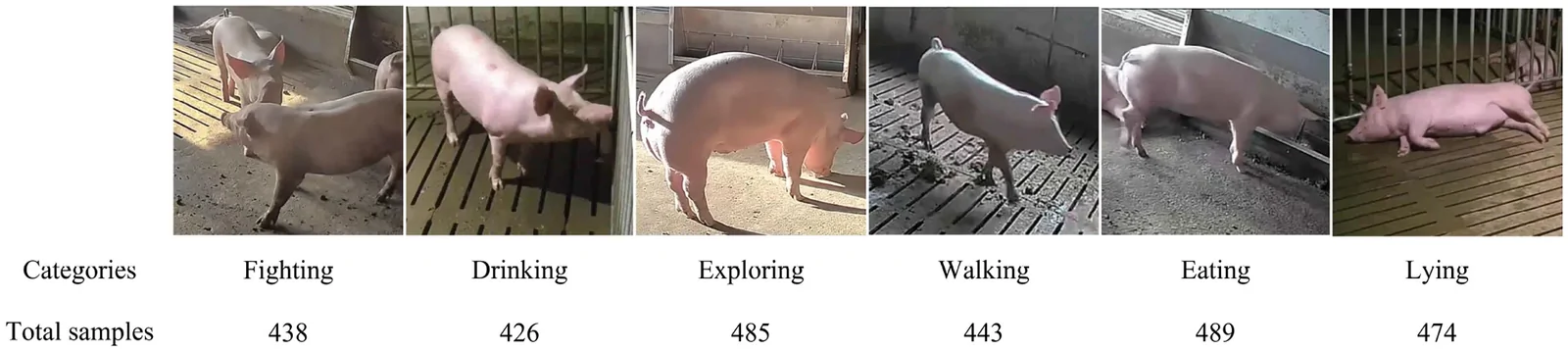
Six categories of pig behavior and their data quantities.

A total of 1.5 TB of raw data was collected, including over 5,000 video files. During preprocessing, raw videos were first manually inspected to remove low-quality, occluded, or behavior-ambiguous samples. Only videos with clearly observable and continuous behaviors were retained. After screening, effective video clips with durations of 5–10 seconds were retained. These clips were then annotated at the clip level by two trained veterinary specialists using the LabelImg software, where each entire video clip was assigned a single behavior label. From this, a dataset was created with six behavior categories (29): Fighting, Drinking, Exploring (30), Walking, Eating, and Lying. These six categories were selected as they represent the most critical indicators of pig welfare and productivity in a livestock setting, covering both social interaction (Fighting) and basic sustenance/rest behaviors (Drinking, Eating, Lying). Each category contained approximately 450 samples, resulting in a total of 2,775 annotated samples. To improve reproducibility and prevent data leakage, dataset splitting was performed at the video-clip level. Clips extracted from the same continuous video sequence were strictly assigned to either the training or test set, and never shared across splits. The dataset was then divided into training and test sets in an 80:20 ratio. Therefore, the dataset provides a reasonable basis for the controlled model comparisons and ablation experiments conducted in this study, while broader cross-farm and cross-environment generalization requires further validation using larger multi-farm datasets. Detailed statistics regarding the number of videos in each category are presented in Figure 2.

### 2.2 MMBs_TransNeXt

**Figure 3:**
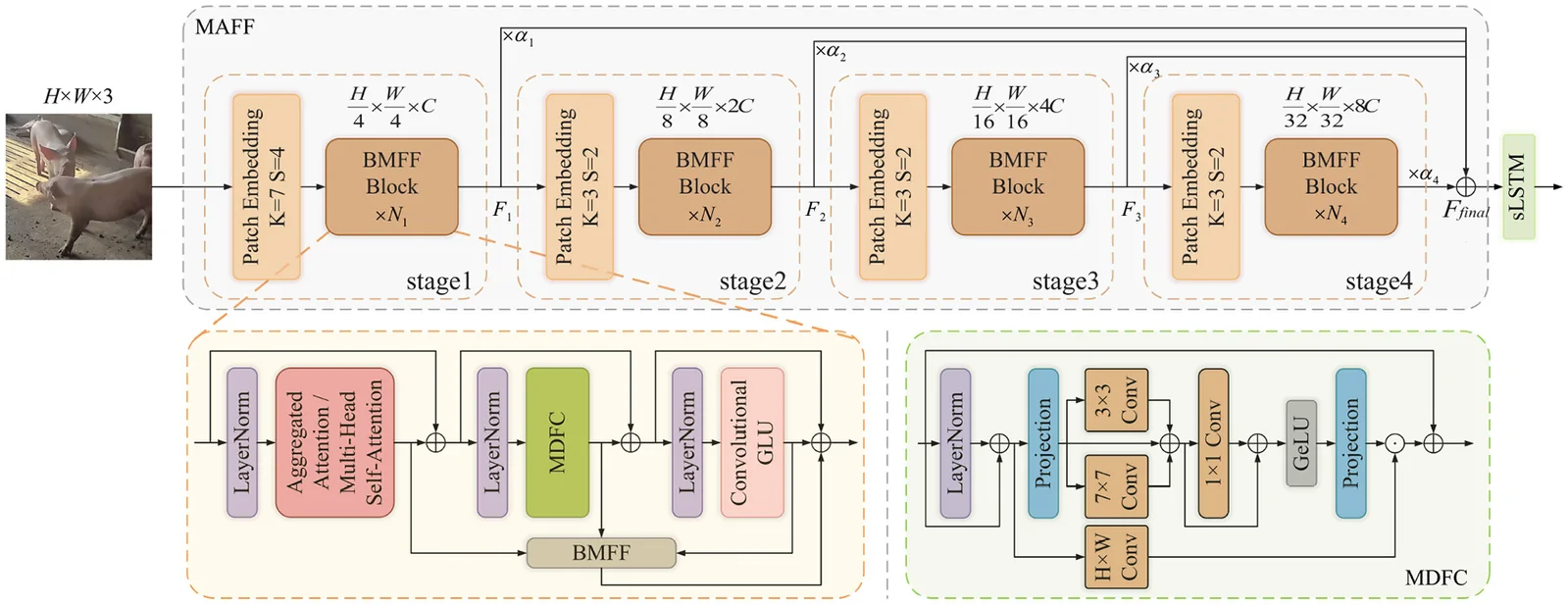
The overall architecture of MMBs_TransNeXt. The network consists of *N* stages. The input is processed by a stem layer. Each stage contains an MDFC module followed by a BMFF mechanism for intra-stage feature refinement. Features from all stages *F*_1_, *F*_2_, *F*_3_, *F*_4_ are adaptively fused by the MAFF mechanism to produce the final spatial feature representation *F*_*final*_. The fused spatial feature representation *F*_*final*_ is processed by sLSTM for temporal modeling.

A novel pig behavior recognition model called MMBs_TransNeXt was proposed, as illustrated in Figure 3. The model consists of three core components: the Multi-stage Adaptive Feature Fusion (MAFF) mechanism, the Multi-scale Detail Fusion Convolution (MDFC) module, and the Bidirectional Modulation Feature Fusion (BMFF) mechanism. The MMBs_TransNeXt was developed to address several challenges in complex scenarios, including the effective fusion of multi-scale features to enhance accuracy, the comprehensive capture of pig behavior information, and the achievement of efficient feature interaction and dynamic adaptation to varied pig environments (31).

The overall architecture of the proposed MMBs_TransNeXt is illustrated in Figure 3. It is designed as a deep network comprising *N* hierarchical stages, each responsible for extracting features at a specific scale. The input video clips are first processed by a stem convolutional layer to generate initial feature maps. The core innovation of our model lies in the synergistic integration of three dedicated mechanisms into this multi-stage backbone:

1. The Multi-stage Adaptive Feature Fusion (MAFF) mechanism (Section 2.2.1) operates across the different stages. It dynamically fuses feature maps *F*_1_, *F*_2_, ..., *F*_*N*_ from all stages to produce a comprehensive representation *F*_*final*_ that preserves both fine-grained details and high-level semantics.

2. The Multi-scale Detail Fusion Convolution (MDFC) module (Section 2.2.2) and the Bidirectional Modulation Feature Fusion (BMFF) mechanism (Section 2.2.3) operate within each stage. They are integrated as the fundamental building block to replace standard convolutional layers. Specifically, the output *F*_*i*_ of the *i*-th stage is computed as *F*_*i*_ = *BMFF*(*MDFC*(*F*_*i*−1_)), where *F*_*i*−1_ is the output from the previous stage. This design ensures that features are refined in detail and enhanced through modulation at every level of the network.

#### 2.2.1 Multi-stage Adaptive Feature Fusion: MAFF

As the inter-stage fusion component shown in Figure 3, the Multi-stage Adaptive Feature Fusion (MAFF) mechanism is designed to address the limited data adaptability of feature fusion strategies across network stages. Traditional methods often rely on static fusion strategies, such as fixed-weight Concatenation or weighted summation, to tackle feature fusion rigidity. However, these strategies lack data-specific adaptability, preventing them from dynamically adjusting the contributions of multi-scale features and potentially obscuring discriminative information.

The MAFF mechanism enables dynamic fusion through hierarchical weight-learning modules, which facilitates more accurate recognition of pig behavior. The MMBs_TransNeXt model consists of *N* stages, with feature maps denoted as $F_{i}\in {\mathbb{R}}^{B\times C_{i}\times H_{i}\times W_{i}}$ produced at stage *i*∈{0, …, *N*}. Here, *B* represents the batch size, *C* indicates the total number of channels, and *H* and *W* denote the height and width of *F*_*i*_. Before fusion, feature maps from different stages were first aligned to a common feature space in terms of spatial resolution and channel dimension, and the aligned features were then weighted by α_*i*_ to obtain *F*_*final*_. The implementation of the MAFF is described in Equation 1:


$$\begin{array}{l} F_{final}=\overset{N}{\underset{i=1}{\sum }}F_{i}⊙\alpha_{i} \end{array}$$ (1)

where *F*_*final*_ represents the output fused feature. ⊙ denotes element-wise multiplication. α_*i*_ is the stage-adaptive weight for the *i*-th stage feature map *F*_*i*_. The MAFF mechanism effectively addresses shallow-deep feature complementarity. Shallow features like *F*_1_ and *F*_2_ are detail-rich but semantically weak. Deep features like *F*_*N*−1_ and *F*_*N*_ are semantically strong but lose details. Complementary fusion is achieved by MAFF through stage-adaptive weights α_*i*_. This approach facilitates the capture of subtle pig movements while effectively mitigating issues related to feature fusion rigidity. Details regarding the stage-adaptive weights α_*i*_ can be found in Equation 2.


$$\begin{array}{l} \alpha_{i}=Softmax\left(W_{i}F_{i}+b_{i}\right) \end{array}$$ (2)

where *Softmax* function is applied as activation. *W*_*i*_ denotes learnable parameters. *b*_*i*_ represents the bias.

#### 2.2.2 Multi-scale Detail Fusion Convolution: MDFC

**Figure 4:**
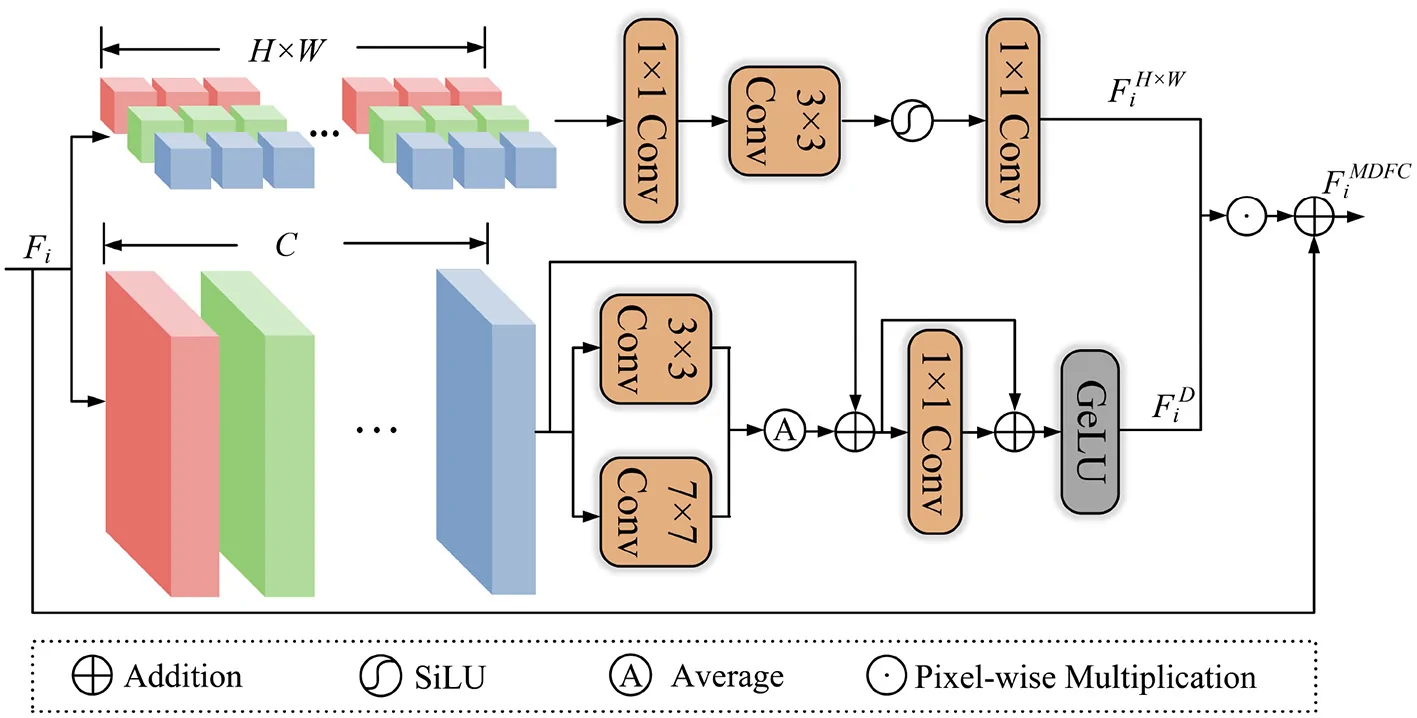
The MDFC architecture diagram.

The Multi-scale Detail Fusion Convolution (MDFC) module serves as the first part of our intra-stage feature refinement block (Figure 3). It is designed to mitigate the issue of feature detail blurring caused by cross-channel interference within a stage, as illustrated in Figure 4. This module comprises two collaborative branches: an HW-dimensional convolution (32) branch and a dual-scale receptive field convolution branch.

Specifically, the feature *F*_*i*_ from the *i*-th stage is output by the MDFC module as $F_{i}^{MDFC}$. This is implemented as shown in Equation 3


$$\begin{array}{l} F_{i}^{MDFC}=\left(F_{i}^{H\times W}⊙F_{i}^{D}\right)+F_{i} \end{array}$$ (3)

where $F_{i}^{H\times W}$ is the output from the HW-dimensional convolution channel branch, which is detailed in Equation 4. $F_{i}^{D}$ is represented as the output through the Dual-scale receptive field convolution channel branch, as specified in Equation 5.

The output of the HW-dimensional convolution channel branch is defined as:


$$\begin{array}{l} F_{i}^{H\times W}={Conv}_{1\times 1}\left(SiLU\left(f_{i}^{H\times W}\right)\right),\;\\ \;f_{i}^{H\times W}={Conv}_{3\times 3}\left({Conv}_{1\times 1}\left(F_{i}\right)\right) \end{array}$$ (4)

where *SiLU* is applied as the activation function. *Conv*_1 × 1_ and *Conv*_3 × 3_ represent convolution operations with kernels of size 1 × 1 and 3 × 3, respectively. The HW-dimensional convolution channel branch prevents cross-channel noise transmission through independent channel operations, thereby enhancing edge detail sharpening.

For the Dual-scale receptive field convolution channel branch, there are two sub-branches: small and large receptive fields. The small receptive field branch captures fine-grained behavioral features using 3 × 3 channel convolution, while the large receptive field branch captures global contour features through 7 × 7 channel convolution. This design allows for cross-channel fusion, preventing structural information loss that can occur due to channel isolation.

The output for the Dual-scale receptive field convolution channel branch is defined as follows:


$$\begin{array}{c} F_{i}^{D}=GELU\left(f_{i}^{D}+{Conv}_{1\times 1}\left(f_{i}^{D}\right)\right),\;\\ f_{i}^{D}=F_{i}+avg\left({Conv}_{3\times 3}\left(F_{i}\right)+{Conv}_{7\times 7}\left(F_{i}\right)\right) \end{array}$$ (5)

where *GELU* serves as the activation function. *avg* denotes the averaging operation, and *Conv*_7 × 7_ indicates a convolution with a kernel size of 7 × 7.

Specifically, the 3 × 3 branch mainly captures fine local behavioral details such as head movement, mouth movement, limb motion, and local body-edge changes, whereas the 7 × 7 branch captures broader spatial cues such as body contour, body orientation, and head–trunk–limb posture relationships.

#### 2.2.3 Bidirectional Modulation Feature Fusion: BMFF

**Figure 5:**
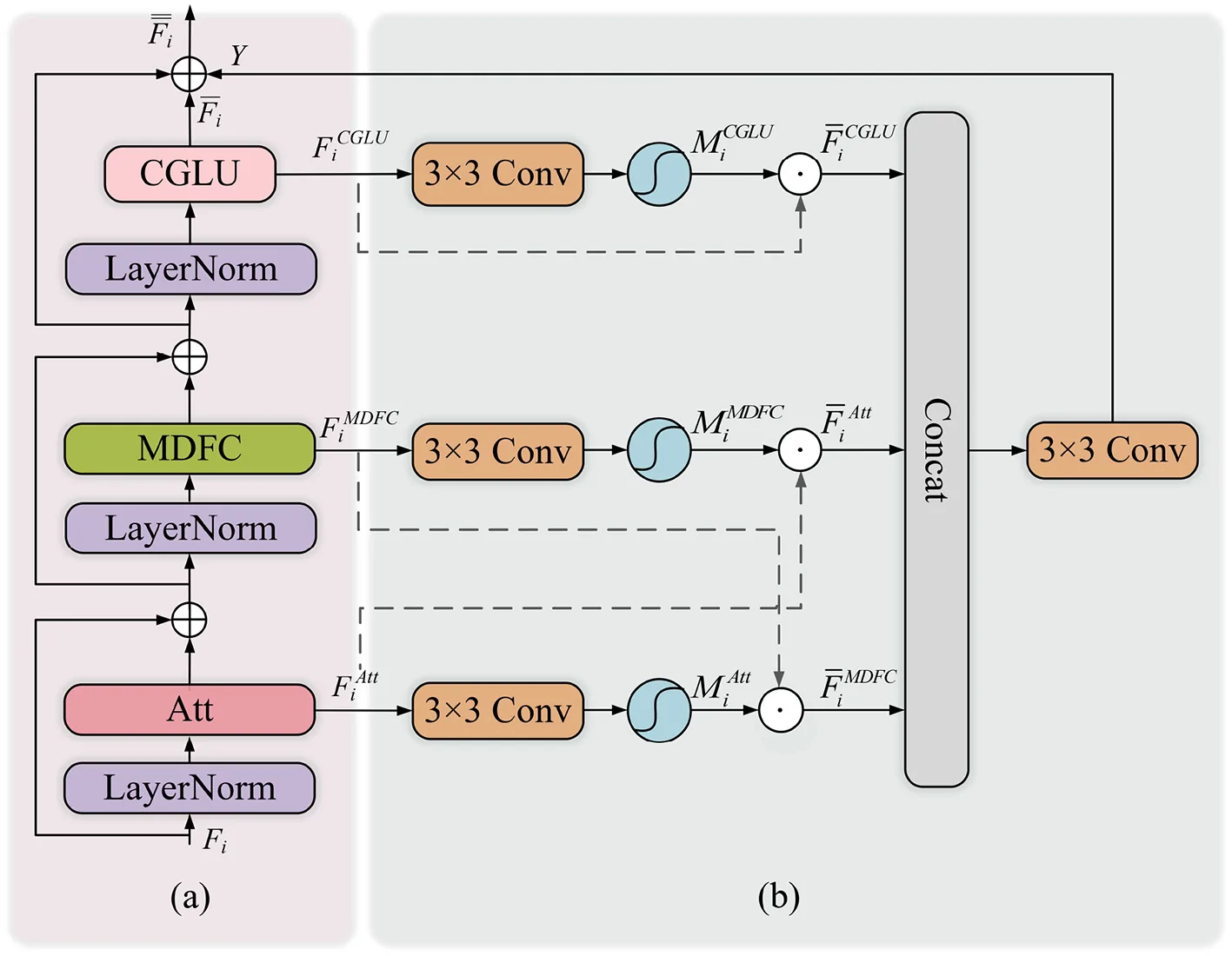
The BMFF architecture diagram. **(a)** Self-modulation branch. **(b)** Cross-modulation branch.

Following the MDFC module within each stage (Figure 3), the Bidirectional Modulation Feature Fusion (BMFF) mechanism is proposed to enhance cross-level feature interactions. It takes the output of MDFC as its primary input. However, this limitation makes it challenging to fully integrate the features extracted at various levels, ultimately hindering the model's performance in recognizing pig behavior. To address this issue, we propose a Bidirectional Modulation Feature Fusion (BMFF) mechanism. This mechanism constructs cross-component interaction paths through three modules: Aggregated Attention (27)/Multi-Head Self-Attention (33) (Att), Multi-scale Detail Fusion Convolution (MDFC), and Convolutional Gated Linear Unit (CGLU) (28). The details of the BMFF architecture are illustrated in Figure 5.

The BMFF processes the input feature map *F*_*i*_ through two pathways: a self-modulation branch and a cross-modulation branch. The self-modulation branch applies sequential transformations to *F*_*i*_ to produce a base feature ${\bar{F}}_{i}$, as shown in Figure 5a. The cross-modulation branch then utilizes intermediate outputs from this process to generate a modulated feature Y via cross-modulation operations, as depicted in Figure 5b. Finally, the output feature map ${\bar{\bar{F}}}_{i}$ is obtained by summing ${\bar{F}}_{i}$ and the modulated *Y*, as described in Equation 6:


$$\begin{array}{l} {\bar{\bar{F}}}_{i}=Y+{\bar{F}}_{i}=BMFF\left(F_{i}\right) \end{array}$$ (6)

The calculation process of the self-modulation branch can be formally expressed in Equations 7 and 8:


$$\begin{array}{l} {\bar{F}}_{i}=F_{i}^{CGLU}=CGLU\left(F_{i}^{MDFC}+F_{i}\right) \end{array}$$ (7)

$$\begin{array}{l} F_{i}^{MDFC}=MDFC\left(F_{i}^{Att}+F_{i}\right),F_{i}^{Att}=Att\left(F_{i}\right) \end{array}$$ (8)

where $F_{i}^{CGLU}$ is denoted as the output feature map from the CGLU module. $F_{i}^{MDFC}$ represents the output feature map from the MDFC module. $F_{i}^{Att}$ is defined as the output feature map from the Att module.

The computational process of the cross-modulation branch is expressed as Equation 9.


$$\begin{array}{l} Y={Conv}_{fuse}\left(Concat\left({\bar{F}}_{i}^{Att},{\bar{F}}_{i}^{MDFC},{\bar{F}}_{i}^{CGLU}\right)\right) \end{array}$$ (9)

where *Conv*_*fuse*_ is implemented as a 3 × 3 convolutional layer, which is used to fuse the Concatenated features. *Concat* denotes channel-wise Concatenation. $F_{i}^{Att}$, $F_{i}^{MDFC}$, and $F_{i}^{CGLU}$ represent the weighted features from the Att, MDFC, and CGLU modules, respectively. Definitions are provided in Equation 10.


$$\begin{array}{l} {\bar{F}}_{i}^{Att}=M_{i}^{MDFC}⊙F_{i}^{Att},\\ {\bar{F}}_{i}^{MDFC}=M_{i}^{Att}⊙F_{i}^{MDFC},\\ {\bar{F}}_{i}^{CGLU}=M_{i}^{CGLU}⊙F_{i}^{CGLU} \end{array}$$ (10)

where $M_{i}^{Att}$, $M_{i}^{MDFC}$ and $M_{i}^{CGLU}$ act as modulation weights for $F_{i}^{Att}$, $F_{i}^{MDFC}$, and $F_{i}^{CGLU}$.

Equation 10 embodies the core modulation mechanism of BMFF: cross-modulation and self-modulation. For cross-modulation, the spatial attention distribution is focused by the Att module, while the MDFC module emphasizes multi-scale texture fusion. The output features from both modules are weighted by modulation weights $M_{i}^{Att}$ and $M_{i}^{MDFC}$ generated from each other. Spatial attention provides weighted guidance for multi-scale features, which in turn enhances the representation of details by the attention region. For self-modulation, the CGLU acts as a gating unit, controlling the information flow. It weights the output features via self-generated $M_{i}^{CGLU}$. Gating intensity is adaptively adjusted to avoid modulation interference from the Att and MDFC modules, ensuring stability and maintaining feature information flow efficiency. The mutual collaboration between the two modulations enhances cross-feature complementarity and gating control, ultimately improving the representation capability of pig behavior.

The generation of the modulation weights is defined in Equation 11:


$$\begin{array}{l} M_{i}^{Att}=Sigmoid\left({Conv}_{d0}\left(F_{i}^{Att}\right)\right)\\ M_{i}^{MDFC}=Sigmoid\left({Conv}_{d1}\left(F_{i}^{MDFC}\right)\right)\\ M_{i}^{CGLU}=Sigmoid\left({Conv}_{d2}\left(F_{i}^{CGLU}\right)\right) \end{array}$$ (11)

where *Conv*_*d*0_, *Conv*_*d*1_ and *Conv*_*d*2_ are implemented as three separate convolutional layers with 3 × 3 kernel sizes. This process extracts information from the corresponding module outputs, generating the modulation weights *M*.

## 3 Experiment and analysis

To validate the effectiveness of MMBs_TransNeXt model, we conducted systematic experiments. We evaluated the MMBs_TransNeXt model on a system equipped with 125 GB of RAM, an Intel i7-7800X CPU operating at 3.50 GHz, and an NVIDIA TITAN Xp GPU with 12 GB of GDDR5X memory. The Ubuntu 20.04.6 LTS environment ran Python 3.12.4 with core libraries PyTorch 2.1.2, Transformers 4.35.0, and NumPy 2.0.1. For each video clip, 24 frames were uniformly sampled and resized to 224 × 224 pixels; after spatial feature fusion, the sLSTM module was used to model temporal dependencies among the sampled frames. The input tensor dimensions were [batch_size, num_frames, channels, height, width]=[4, 24, 3, 224, 224]. Training was performed using the Adam optimizer with a batch size of 4, an initial learning rate of 0.001, for over 200 epochs. The model produces classification outputs of dimensions [4,6], corresponding to the batch size and number of classes. MMBs TransNeXt performs six-class pig behavior recognition at the video-clip level. Each input video clip corresponds to one behavior label, and the model does not output object locations or bounding-box coordinates.

We conducted a series of experiments. This section includes: the overall performance verification of the proposed MMBs_TransNeXt; evaluating the effectiveness of the Multi-stage Adaptive Feature Fusion (MAFF); evaluating the effectiveness assessment and structural optimization of the Multi-scale Detail Fusion Convolution (MDFC); evaluating the effectiveness of the Bidirectional Modulation Feature Fusion (BMFF), and an ablation study.

### 3.1 Evaluation of different models

To enhance pig behavior recognition performance, we propose a model called MMBs_TransNeXt. We compared mainstream models, including ViT, Swin Transformer, ConvNeXt, Efficient ViT (34), CNN-LSTM (35) and TransNeXt. The results of this comparison are presented in Table 1.

**Table 1:** Comparison results of MMBs_TransNeXt with different transformer models.

Model	Accuracy (%)	Loss	Precision (%)	Recall (%)	F1-Score (%)	Params	FLOPs
ViT	83.21	0.8597	83.79	83.44	83.99	48.7M	9.4G
Swin Transformer	90.51	0.4902	90.88	90.32	90.40	42.5M	8.7G
ConvNeXt	92.52	0.4880	92.48	92.52	92.47	28M	4.5G
Efficient ViT	90.51	0.4616	90.57	90.57	90.50	7.5M	1.5G
CNN-LSTM	90.00	0.4291	90.14	89.92	89.82	15.0M	2.7G
TransNeXt	92.88	0.4777	92.87	92.87	92.86	13.2M	2.7G
**MMBs_TransNeXt (Ours)**	**95.77**	**0.2301**	**95.78**	**95.59**	**95.64**	15.6M	2.8G

Bold values indicate the best results for the corresponding evaluation metrics.

As shown in Table 1, MMBs_TransNeXt consistently outperforms the other models across all key metrics. It achieves an accuracy of 95.77% and a loss value of 0.2301, demonstrating its efficiency. Moreover, the MMBs_TransNeXt exhibits a precision of 95.78%, a recall of 95.59%, and an F1-score of 95.64%. When compared to mainstream Transformer models, the MMBs_TransNeXt shows significant improvements in several core metrics. Specifically, it records a 12.56 percentage point increase in accuracy and a 73.23% reduction in loss compared to the Vision Transformer (ViT). Precision, recall, and F1-score are also enhanced by 11.99, 12.15, and 11.65 percentage points, respectively. In comparison to Swin Transformer, the MMBs_TransNeXt achieves 5.26 percentage points higher accuracy and 53.06% lower loss, along with improvements of 4.90 percentage points in precision, 5.27 percentage points in recall, and 5.24 percentage points in F1-score. Against the ConvNeXt model, the MMBs_TransNeXt shows a 3.25 percentage point improvement in accuracy and a 52.85% reduction in loss, with increases of 3.30 percentage points in precision, 3.07 percentage points in recall, and 3.17 percentage points in F1-score. For Efficient ViT, MMBs_TransNeXt achieves a 5.26 percentage point higher accuracy, 50.15% lower loss, 5.21 percentage points better precision, 5.02 percentage points improved recall, and 5.14 percentage points higher F1-score. Against CNN-LSTM, MMBs_TransNeXt records a 5.77 percentage point accuracy increase, 46.37% loss reduction, 5.64 percentage points precision improvement, 5.67 percentage points recall enhancement, and 5.82 percentage points F1-score gain. Even when compared to TransNeXt, it achieves 2.89 percentage points higher accuracy and 51.83% lower loss, along with increases of 2.91 percentage points in precision, 2.72 percentage points in recall, and 2.78 percentage points in F1-score.

MMBs TransNeXt contains 15.6M parameters and requires 2.8G FLOPs, reflecting a trade-off between recognition accuracy and computational cost. The current results alone are insufficient to demonstrate its suitability for edge deployment, and its practical deployment performance requires further evaluation on edge devices.

**Figure 6:**
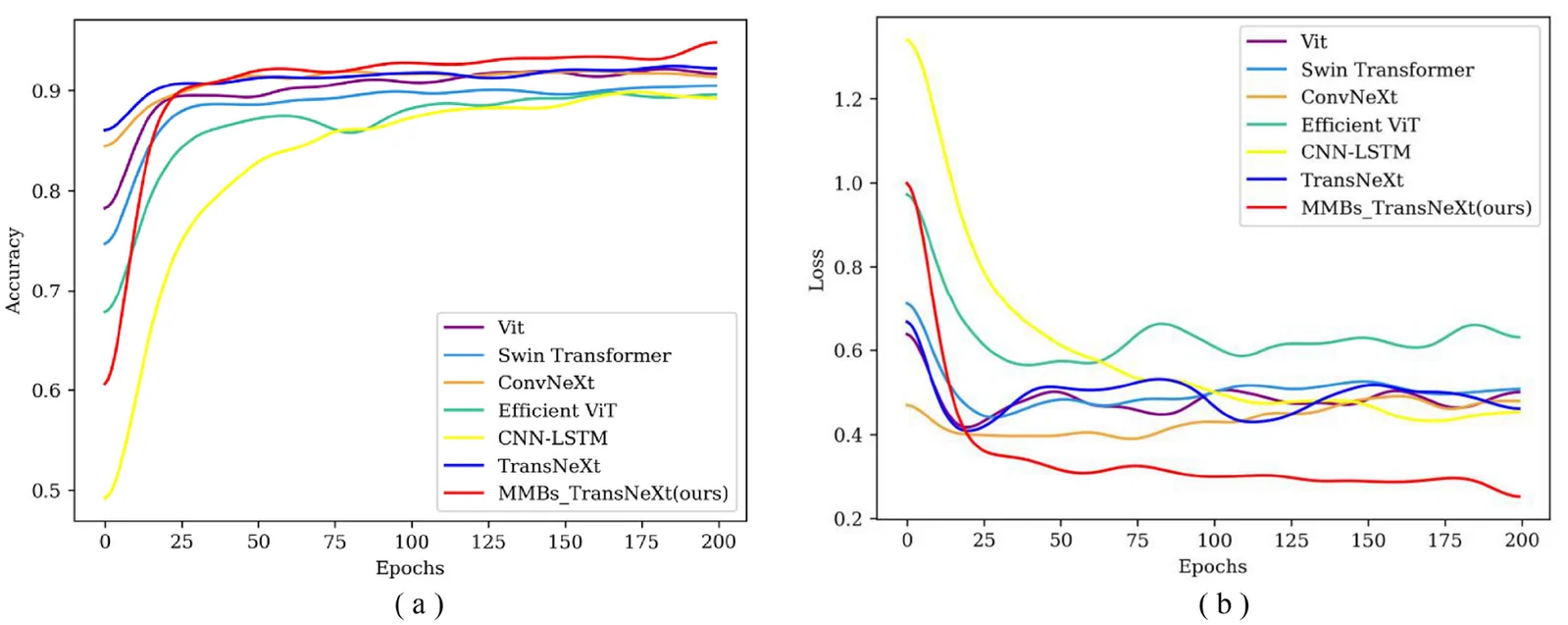
The accuracy and loss curves of pig behavior recognition for different models at various training periods. **(a)** represents accuracy, and **(b)** represents the loss.

To further validate the effectiveness of MMBs_TransNeXt, Figure 6 presents the accuracy and loss curves of all models throughout the training epochs.

An analysis of Figure 6 demonstrates that MMBs_TransNeXt outperforms other models in terms of accuracy and loss. Specifically, Figure 6a shows that MMBs_TransNeXt achieves higher accuracy compared to ViT, Swin Transformer, ConvNeXt, and TransNeXt. Meanwhile, Figure 6b illustrates that MMBs_TransNeXt has the lowest loss among these models. These findings further validate the effectiveness of MMBs_TransNeXt for recognizing pig behavior.

Table 2 highlights the excellent performance of the MMBs_TransNeXt model across six pig behavior categories. The model achieved the highest accuracy in identifying drinking, eating, exploring, lying, and walking behaviors. Although the recognition accuracy of Fighting behavior was lower than that of the top four categories, it still maintained a competitive level. The performance gap in recognizing Fighting behavior can be attributed to limitations of the dataset, particularly video blur, which hinders the model's ability to capture the essential features of pigs. Overall, the results in pig behavior recognition are satisfactory, strongly supporting the model's effectiveness in these tasks.

**Table 2:** Comparison of the recognition performance of different models in six pig behavior categories.

Model	Metric	Category						Avg Prec	Avg Rec	Avg F1
		Drinking	Eating	Fighting	Exploring	Lying	Walking			
ViT	Precision	88.30	91.67	86.05	80.00	94.12	62.62	83.94	83.21	83.28
	Recall	97.65	90.72	85.06	65.98	85.11	76.14			
	F1-Score	92.74	91.19	85.55	72.32	89.39	68.72			
Swin Transformer	Precision	96.47	95.92	94.94	78.95	94.79	84.21	90.77	90.51	90.44
	Recall	96.47	96.91	86.21	92.78	96.81	72.73			
	F1-Score	96.47	96.41	90.36	85.31	95.79	78.05			
ConvNeXt	Precision	93.41	95.96	96.43	90.72	94.44	83.91	92.52	92.52	92.49
	Recall	100.00	97.94	93.10	90.72	90.43	82.95			
	F1-Score	96.59	96.94	94.74	90.72	92.39	83.43			
Efficient ViT	Precision	95.45	97.89	87.64	93.10	91.49	77.89	90.73	90.51	90.55
	Recall	98.82	95.88	89.66	83.51	91.49	84.09			
	F1-Score	97.11	96.88	88.64	88.04	91.49	80.87			
CNN-LSTM	Precision	92.22	93.75	85.26	84.11	97.67	87.84	90.18	89.96	89.87
	Recall	97.65	92.78	93.10	92.78	89.36	73.86			
	F1-Score	94.86	93.26	89.01	88.24	93.33	80.25			
TransNeXt	Precision	96.43	97.87	88.89	90.63	95.79	87.64	92.94	92.88	92.90
	Recall	95.29	94.85	91.95	89.69	96.81	88.64			
	F1-Score	95.86	96.34	90.40	90.16	96.30	88.14			
MMBs_TransNeXt (Ours)	Precision	97.65	97.94	96.55	97.94	96.81	87.76	**95.77**	**95.76**	**95.73**
	Recall	98.81	97.94	92.31	93.14	97.85	94.51			
	F1-Score	98.22	97.94	94.38	95.48	97.33	91.01			

Bold values indicate the best results for the corresponding evaluation metrics.

The superior performance of the MMBs_TransNeXt model in recognizing pig behavior can be attributed to several key aspects. Firstly, the Multi-stage Adaptive Feature Fusion (MAFF) mechanism enhances the representation of important behavioral features in pigs by dynamically adjusting weights across different stages of the network. Secondly, the Multi-scale Detail Fusion Convolution (MDFC) module utilizes a dual-branch architecture that processes both spatial and channel dimensions separately. This approach suppresses cross-channel interference while preserving crucial behavioral details. Lastly, the Bidirectional Modulation Feature Fusion (BMFF) mechanism incorporates both cross-modulation and self-modulation mechanisms, facilitating improved interactions between features at different levels. The improvement is mainly attributed to the complementary effects of MAFF, MDFC, and BMFF, which respectively enhance adaptive stage fusion, local-detail and global-contour representation, and cross-level feature interaction; these mechanisms are especially useful for difficult categories such as Fighting and Walking, where fast movement, overlap, motion blur, and subtle limb or posture changes can easily cause misclassification.

### 3.2 Evaluate the effectiveness of MAFF

To address the rigidity in feature fusion caused by fixed weight allocation in traditional methods, this study introduces a Multi-stage Adaptive Feature Fusion (MAFF) mechanism. MAFF dynamically evaluates the importance of features extracted from different stages of the network and adaptively generates fusion weights. This process enhances the model's ability to capture characteristics of pig behavior.

In this section, we utilize the s_Swin Transformer, s_ConvNeXt, and s_TransNeXt models as baseline models to evaluate the effectiveness of MAFF through controlled experiments. The “s_” prefix indicates that these models incorporate the Scalar Long Short-Term Memory (sLSTM) module (36) into three mainstream backbone networks: Swin Transformer, ConvNeXt, and TransNeXt.

Table 3 demonstrates that utilizing the MAFF mechanism improves the performance of models in terms of both accuracy and loss metrics. Specifically, the three model variants achieved accuracy increases of 0.14, 0.25, and 0.27 percentage points, while losses decreased by 1.01%, 9.20%, and 19.66%, respectively. Notably, the s_ConvNeXt and s_TransNeXt models demonstrated a loss reduction of over 9%, highlighting MAFF's effectiveness in reducing feature fusion noise. By dynamically assessing feature importance at different stages of the network and adaptively assigning fusion weights, MAFF successfully addresses the issue of feature rigidity associated with traditional fixed-weight fusion methods. This mechanism exhibited outstanding performance in the s_TransNeXt model, achieving a record-low loss value of 0.3101 and an accuracy improvement of 0.27 percentage points. This result confirms the enhanced feature extraction capability in complex scenarios through dynamic weight allocation.

**Table 3:** Comparison of different models regarding the utilization of MAFF.

Model	MAFF	Accuracy (%)	Loss
s_Swin Transformer	×	90.88	0.4855
	✓	91.02	0.4806
s_ConvNeXt	×	92.63	0.4758
	✓	92.88	0.4320
s_TransNeXt	×	93.98	0.3860
	✓	**94.25**	**0.3101**

Bold values indicate the best results for the corresponding evaluation metrics.

**Figure 7:**
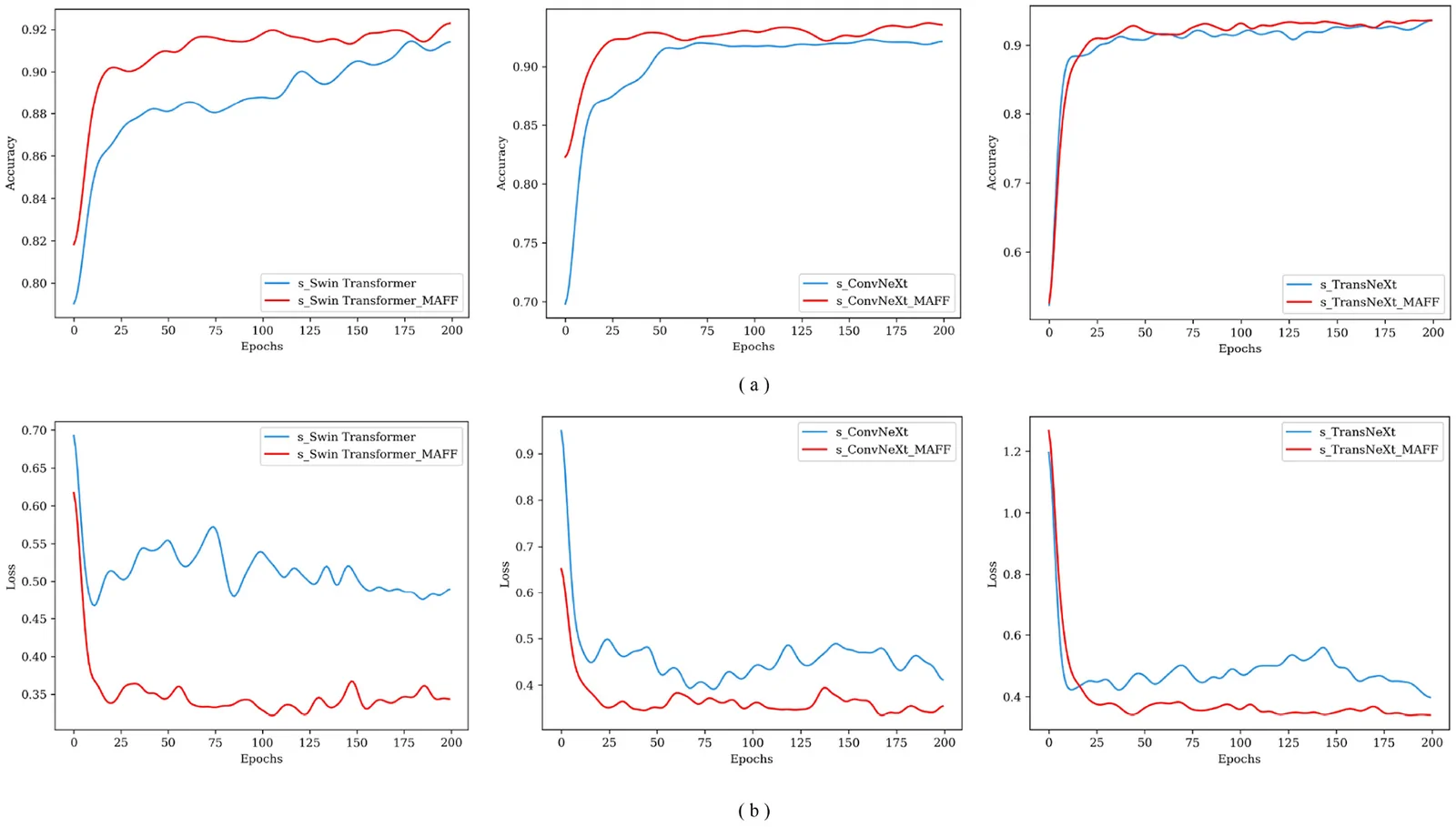
The accuracy and loss rate curves of the models with and without MAFF. **(a)** represents the accuracy, and **(b)** represents the loss.

To further validate the effectiveness of MAFF, Figure 7 compares the accuracy and loss curves of MAFF-integrated models against their baseline counterparts.

As illustrated in Figure 7a, models enhanced with the MAFF mechanism consistently outperform baseline models while maintaining stable accuracy. Figure 7b shows that the loss reduction in MAFF-integrated models follows smoother trajectories and achieves significantly lower loss values compared to the baselines.

In tasks involving pig behavior recognition, models that incorporate the MAFF mechanism show distinct performance advantages. This mechanism facilitates dynamic feature fusion by constructing a hierarchical weight learning module that generates stage-adaptive weights. Such a fusion strategy optimizes the complementary information during feature aggregation and enhances the discriminative representation of critical behavioral features through adaptive weighting. This process leads to more distinguishable expressions of behavioral patterns. Experimental comparisons indicate that models equipped with MAFF achieve higher recognition accuracy in complex tasks related to pig behavior than their non-MAFF counterparts.

### 3.3 The effectiveness evaluation and structural optimization of MDFC

#### 3.3.1 Evaluate the effectiveness of MDFC

To address the issue of behavioral detail blurring caused by cross-channel information interference, we propose a Multi-scale Detail Fusion Convolution (MDFC) module. This module utilizes a dual-branch parallel architecture to improve feature extraction capabilities across various dimensions, focusing specifically on local details and global features of pig behaviors.

We selected models incorporating the MAFF mechanism from Section 3.3, including Ms_Swin Transformer, Ms_ConvNeXt, and Ms_TransNeXt, as baseline models. We systematically compared the performance of the Mona (37) and MDFC modules with their corresponding baselines to evaluate the impact of the MDFC module on behavior recognition capabilities. The experimental results are presented in Table 4.

**Table 4:** Performance evaluation of MDFC enhanced models.

Model	Mona	MDFC	Accuracy (%)	Loss
Ms_Swin Transformer	×	×	91.02	0.4806
	✓	×	93.37	0.3464
	×	✓	93.60	0.3330
Ms_ConvNeXt	×	×	92.88	0.4320
	✓	×	94.16	0.2769
	×	✓	94.36	0.2610
Ms_TransNeXt	×	×	94.25	0.3101
	✓	×	95.30	0.3080
	×	✓	**95.62**	**0.2326**

Bold values indicate the best results for the corresponding evaluation metrics.

Experimental results consistently show that all modified models outperform baseline configurations. In the Ms_Swin Transformer, the integration of Mona resulted in a 2.35 percentage point increase in accuracy and a 27.92% reduction in loss. The use of MDFC further improved the accuracy by 2.58 percentage points and decreased the loss by 30.71% compared to the baseline. For the Ms_ConvNeXt model, the Mona integration led to a 1.28 percentage point gain in accuracy and a 35.88% reduction in loss. In contrast, MDFC enhanced the accuracy by 1.48 percentage points (from 92.88% to 94.36%) and reduced the loss to 0.2610. Meanwhile, in the Ms_TransNeXt framework, the Mona integration improved accuracy by 1.05 percentage points with a slight loss reduction of 0.65%; the MDFC achieved an accuracy improvement of 1.37 percentage points (from 94.25% to 95.62%) and a significant 24.48% reduction in loss compared to Mona.

The MDFC module adopts a heterogeneous dual-branch design to enhance local detail extraction and global contour capture, effectively mitigating behavioral detail blurring induced by cross-channel interference. When compared to Mona, the MDFC demonstrates better loss optimization across all three architectures, with Ms_TransNeXt achieving a notable 24.51% reduction in loss. Importantly, this improvement comes while maintaining consistent gains in accuracy. These results validate the effectiveness of the MDFC's multi-scale fusion strategy in capturing the behavioral characteristics of pigs.

**Figure 8:**
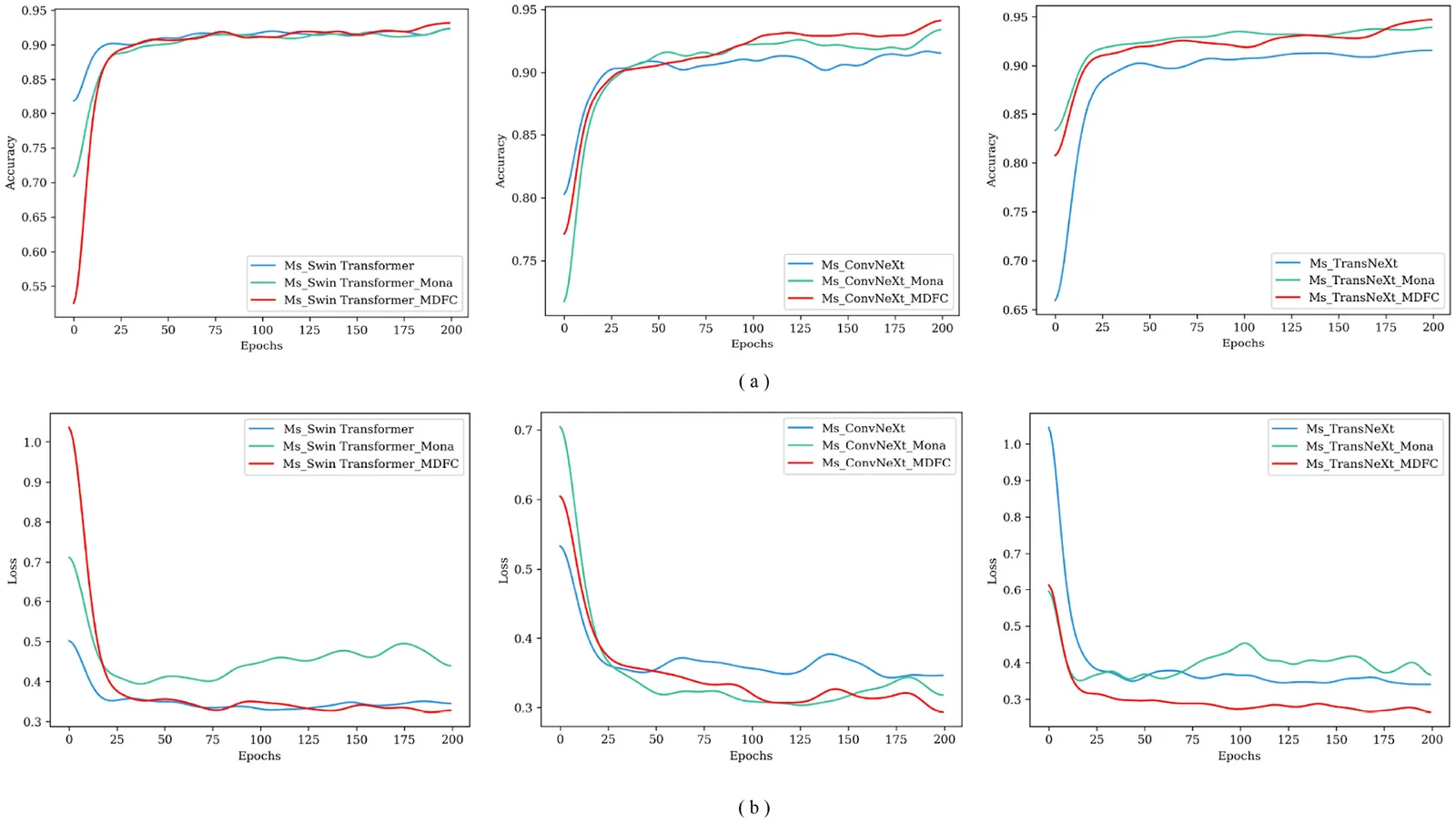
Comparison of the accuracy and loss curves of the model with and without the MDFC. **(a)** represents the accuracy, and **(b)** represents the loss.

To further evaluate the effectiveness of MDFC, Figure 8 displays comparative analyses of accuracy and loss curves between models that use MDFC and those that do not.

Figure 8a shows that models equipped with the MDFC unit consistently demonstrate improved accuracy compared to models without it. As illustrated in Figure 8b, the models using MDFC achieve the lowest loss values. These findings confirm the effectiveness of MDFC in pig behavior recognition tasks.

The performance improvement attributed to MDFC arises from its dual heterogeneous branches. The HW-dimensional convolution branch reduces interference while enhancing local details, and the Dual-scale receptive field channel branch captures fine-grained features through a small receptive field as well as global contours through a larger receptive field. This design significantly strengthens the feature representation capabilities of the model.

The MDFC module integration in pig behavior recognition models outperforms Mona, which prioritizes general visual task adaptation and computational efficiency. By specifically targeting feature interference through its dual-branch architecture, MDFC achieves superior detail and global feature extraction, ultimately resulting in enhanced model performance.

#### 3.3.2 Evaluate the impact of different branch combinations in the MDFC

To systematically assess the impact of various branch combinations within the MDFC module on model performance, Ms_TransNeXt is employed as the baseline model. We conducted ablation experiments to analyze how different configurations of the HW-dimensional convolution channel branch and the Dual-scale receptive field convolution channel branches, using 3 × 3, 5 × 5, and 7 × 7 kernels, affect accuracy and loss. Detailed results can be found in Table 5.

**Table 5:** Comparison of Msmodel performance for different branch combinations.

Index	HW-branch	Dual-scale branch			Accuracy (%)	Loss
	H×W	3×3	5×5	7×7		
1	×	×	×	×	94.25	0.3101
2	×	✓	✓	✓	95.30	0.3080
3	✓	×	×	×	89.42	0.4332
4	✓	✓	✓	×	91.79	0.3588
5	✓	×	✓	✓	95.44	0.2392
6	✓	✓	×	✓	**95.62**	**0.2326**
7	✓	✓	✓	✓	90.00	0.4500

Bold values indicate the best results for the corresponding evaluation metrics.

Compared to the baseline model Ms_TransNeXt (Index 1), the introduction of the Dual-scale branch with 3 × 3, 5 × 5, and 7 × 7 kernels (Index 2) resulted in an accuracy improvement of 1.05 percentage points and a reduction in loss of 0.65%. This shows that the multi-scale kernels in the Dual-scale branch are effective in capturing diverse aspects of pig behavior, compensating for the baseline's limited ability to represent features.

However, when only the HW-branch is introduced (Index 3), there is a significant drop in accuracy of 4.83 percentage points, and the loss increased by 39.70% compared to Index 1. This indicates that relying solely on the HW-branch is insufficient for capturing multi-scale behavioral features, resulting in significant deficiencies in feature representation. Combining the HW-branch with 3 × 3 and 5 × 5 kernels in the Dual-scale branch (Index 4) resulted in a 2.46 percentage point decrease in accuracy and a 15.70% increase in loss compared to Index 1. Although smaller kernels helped capture local details, the lack of large-scale (7 × 7) coverage limited the perception of global information, leading to reduced performance.

In contrast, combining the HW-branch with 5 × 5 and 7 × 7 kernels (Index 5) achieved a 1.19 percentage point increase in accuracy and a 22.86% decrease in loss compared to Index 1. The medium and large kernels in the Dual-scale branch complemented the HW-branch's ability to extract details, enhancing the precision of feature representation.

Additionally, combining the HW-branch with 3 × 3 and 7 × 7 kernels (Index 6) resulted in a 1.37 percentage point improvement in accuracy and a 25.00% reduction in loss compared to Index 1. The 3 × 3 kernel effectively captured fine local features, while the 7 × 7 kernel covered broader spatial information. This synergy with the HW-branch maximized the benefits of multi-scale feature fusion.

Finally, activating both the HW-branch and the Dual-scale branch simultaneously with all kernels (Index 7) resulted in a 4.25 percentage point drop in accuracy and a 45.11% increase in loss compared to Index 1. The excessive stacking of branches led to feature redundancy and interference caused by overlapping receptive fields, which reduced fusion efficiency.

The optimal configuration for the MDFC is identified as combining the HW-branch with 3 × 3 and 7 × 7 kernels in the Dual-scale branch. This setup effectively addressed the blurring of behavior details caused by cross-channel interference, leveraging the synergistic effects of sharpening details through the HW-branch while providing complementary local and global coverage with the 3 × 3 and 7 × 7 kernels. As a result, this configuration achieved the best performance improvement.

### 3.4 Evaluate the effectiveness of BMFF

To address the issue of pig behavior feature loss caused by insufficient cross-level feature interaction capabilities, the Bidirectional Modulation Feature Fusion (BMFF) mechanism is proposed in this study. Through the synergistic effect of cross-modulation and self-modulation, the model's capacity for cross-level feature interaction is enhanced, thereby improving the accuracy and robustness of pig behavior recognition. Three top-performing improved models incorporating MDFC, including MMs_Swin Transformer, MMs_ConvNeXt, and MMs_TransNeXt, as introduced in Section 3.3.1, are employed as baselines. Detailed experimental results are presented in Table 6.

**Table 6:** Comparison results of different models for whether to use BMFF.

Model	BMFF	Accuracy (%)	Loss
MMs_Swin Transformer	×	93.60	0.3330
	✓	93.80	0.2910
MMs_ConvNeXt	×	94.16	0.2691
	✓	94.36	0.2610
**MMs_TransNeXt**	×	95.62	0.2326
	✓	**95.77**	**0.2301**

Bold values indicate the best results for the corresponding evaluation metrics.

Experimental results show that the performance of all three models improved after the introduction of the BMFF mechanism. The accuracy of the MMs_Swin Transformer increased by 0.20 percentage points, along with a 12.61% reduction in loss. For MMs_ConvNeXt, accuracy improved by 0.20 percentage points, accompanied by a 3.01% decrease in loss. MMs_TransNeXt demonstrated a 0.15 percentage point increase in accuracy and a 1.07% reduction in loss. Overall, the synergistic effect of cross-modulation and self-modulation in BMFF effectively enhanced the models' cross-level feature interaction capabilities. This mechanism showed consistent improvements across different base architectures, indicating stability in both accuracy enhancement and loss convergence.

**Figure 9:**
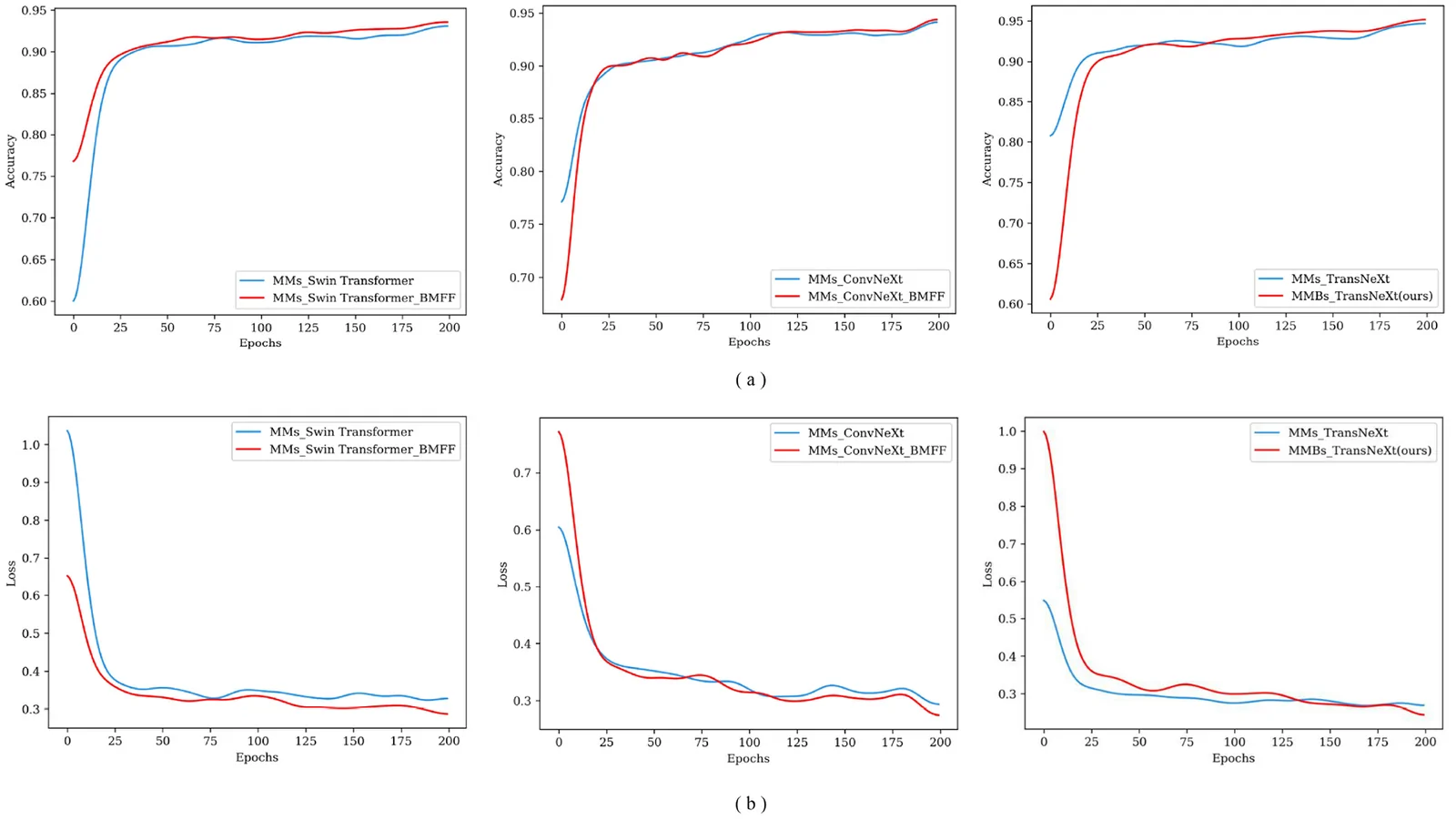
The accuracy and loss rate curves of the models with and without the BMFF in identifying the behaviors of pigs. **(a)** represents the accuracy, and **(b)** represents the loss.

To further validate the effectiveness of BMFF in these models, we compared the variation curves of accuracy and loss values between the baseline models and the BMFF-enhanced models, as shown in Figure 9. In Figure 9a, we observed consistent accuracy improvements in models that adopted BMFF compared to those that did not. Additionally, models incorporating BMFF achieved the lowest loss values, as depicted in Figure 9b.

The BMFF mechanism boosts the performance of the MMs_Swin Transformer, MMs_ConvNeXt, and MMs_TransNeXt architectures, primarily owing to its powerful feature modulation capability. By integrating cross-modulation and self-modulation, BMFF optimizes feature generation and fusion via cross-level interactions. Cross-modulation enhances the complementarity between features of different levels, such as shallow details and deep semantics. This is achieved through cross-level interactions. On the other hand, self-modulation dynamically weights features within a single level, suppressing redundancy while amplifying critical information. The culmination of these processes results in the Att-MDFC-CGLU channel modulation model, which facilitates cross-component feature interaction. The introduction of BMFF significantly improves the model's adaptability to dynamic scenes, leading to substantial gains in pig behavior recognition performance.

### 3.5 Ablation experiment

To systematically evaluate the effectiveness of each module, a series of ablation experiments is conducted in this section. s_TransNeXt is selected as the baseline model. These experiments are designed to analyze the performance contributions of the Multi-stage Adaptive Feature Fusion (MAFF), the Multi-scale Detail Fusion Convolutional (MDFC), and the Bidirectional Modulation Feature Fusion (BMFF) under various combinations.

According to Table 7, when the MAFF is introduced individually (Index 2), the model exhibits further improvements compared to the baseline (Index 1). Specifically, accuracy increases by 0.27 percentage points, and loss decreases by 19.66%. The MAFF mechanism can dynamically adjust fusion weights based on the characteristics of the input, thereby strengthening the representation of pig behavior features.

**Table 7:** Comparison of model performance with different module combinations.

Index	MAFF	MDFC	BMFF	Accuracy (%)	Loss
1	×	×	×	93.98	0.3860
2	✓	×	×	94.25	0.3101
3	✓	✓	×	95.62	0.2326
4	✓	✓	✓	**95.77**	**0.2301**

Bold values indicate the best results for the corresponding evaluation metrics.

Subsequently, the introduction of the MDFC module (Index 3) on top of the previous model leads to an additional accuracy increase of 1.37 percentage points and a loss decrease of 24.99% compared to Index 2. The MDFC module features a dual-branch structure that enhances the model's ability to extract and express the details of pig behavior, further optimizing performance.

Finally, after incorporating the BMFF mechanism (Index 4) into the model developed in Index 3, the MMBs_TransNeXt model achieves optimal performance. Compared to the baseline model, accuracy increases by 1.79 percentage points, and loss decreases by 40.39%. The combination of the MAFF, MDFC, and BMFF modules maximizes the complementary strengths of each component. The MAFF adjusts fusion weights dynamically according to input characteristics, enhancing the representation capability of pig behavior features. The MDFC leverages its dual-branch architecture to sharpen the representation of pig behavior details. Lastly, the BMFF improves the feature representation through cross-module interactions using modulation strategies. Overall, the MMBs_TransNeXt model significantly enhances the accuracy in capturing pig behavior features, achieving a dual optimization of both accuracy and loss values, especially in complex scenarios.

## 4 Conclusions

In this study, we propose MMBs_ TransNeXt, a novel deep learning architecture for accurate and robust pig behavior recognition in precision livestock farming. This model is constructed as a unified hierarchical and adaptive feature learning framework, which effectively overcomes the deficiencies of rigid feature fusion, insufficient spatial detail retention, and weak cross-level feature interaction in existing approaches.

Specifically, our method employs a Multi-stage Adaptive Feature Fusion (MAFF) mechanism to dynamically integrate semantic and fine-grained information across different network stages. It further enhances the representation of subtle behavioral characteristics through a dedicated Multi-scale Detail Fusion Convolution (MDFC) module. In addition, a Bidirectional Modulation Feature Fusion (BMFF) mechanism is introduced to facilitate effective information interaction and feature complementarity among multi-level representations.

Comprehensive experimental results on a real-world pig behavior dataset confirm that MMBs_TransNeXt achieves state-of-the-art performance with 95.77% accuracy, substantially outperforming mainstream CNN and Transformer baselines. These results validate the effectiveness and superiority of the proposed framework in complex farming environments.

For future work, we will expand multi-scene datasets to strengthen cross-environment generalization and develop lightweight versions for edge deployment. This research provides a solid technical foundation for intelligent visual monitoring, welfare evaluation, and refined management in modern precision pig farming systems.

## Data Availability

The raw data supporting the conclusions of this article will be made available by the authors, without undue reservation.
